# Mood Dimensions Show Distinct Within-Subject Associations With Non-exercise Activity in Adolescents: An Ambulatory Assessment Study

**DOI:** 10.3389/fpsyg.2018.00268

**Published:** 2018-03-07

**Authors:** Elena D. Koch, Heike Tost, Urs Braun, Gabriela Gan, Marco Giurgiu, Iris Reinhard, Alexander Zipf, Andreas Meyer-Lindenberg, Ulrich W. Ebner-Priemer, Markus Reichert

**Affiliations:** ^1^Mental mHealth Lab, Department of Sports and Sports Science, Karlsruhe Institute of Technology, Karlsruhe, Germany; ^2^Department of Psychiatry and Psychotherapy, Central Institute of Mental Health, Medical Faculty Mannheim, Heidelberg University, Mannheim, Germany; ^3^Division of Biostatistics, Central Institute of Mental Health, Medical Faculty Mannheim, Heidelberg University, Mannheim, Germany; ^4^GIScience Research Group, Institute of Geography, Heidelberg University, Heidelberg, Germany

**Keywords:** ambulatory assessment, ecological momentary assessment, mood, affective states, physical activity, non-exercise activity, accelerometry, adolescents

## Abstract

Physical activity is known to preserve both physical and mental health. However, the physical activity levels of a large proportion of adolescents are insufficient. This is critical, since physical activity levels in youth have been shown to translate into adulthood. Whereas in adult populations, mood has been supposed to be one important psychological factor that drives physical activity in everyday life, this issue has been poorly studied in adolescent populations. Ambulatory Assessment is the state-of-the-art approach to investigate how mood and non-exercise activity fluctuate within persons in everyday life. Through assessments in real time and real life, this method provides ecological validity, bypassing several limitations of traditional assessment methods (e.g., recall biases). To investigate whether mood is associated with non-exercise activity in adolescents, we equipped a community-based sample comprising 113 participants, aged 12–17 years, with GPS-triggered e-diaries querying for valence, energetic arousal, and calmness, and with accelerometers continuously measuring physical activity in their everyday lives for 1 week. We excluded all acceleration data due to participants' exercise activities and thereafter we parameterized non-exercise activity as the mean value across 10-min intervals of movement acceleration intensity following each e-diary prompt. We used multilevel analyses to compute the effects of the mood dimensions on non-exercise activity within 10-min intervals directly following each e-diary prompt. Additionally, we conducted explorative analyses of the time course of the effects, i.e., on different timeframes of non-exercise activity up to 300 min following the mood assessment. The results showed that valence (*p* < 0.001) and energetic arousal (*p* < 0.001) were positively associated with non-exercise activity within the 10 min interval, whereas calmness (*p* < 0.001) was negatively associated with non-exercise activity. Specifically, adolescents who felt more content, full of energy, or less calm were more physically active in subsequent timeframes. Overall, our results demonstrate significant associations of mood with non-exercise activity in younger ages and converge with the previously observed association between mood and physical activity in adults. This knowledge on distinct associations of mood-dimensions with non-exercise activity may help to foster physical activity levels in adolescents.

## Introduction

In most European countries, less than half of adolescents meet the World Health Organization's physical activity recommendations (WHO, 2011; van Hecke et al., 2016). This is critical since low levels of physical activity during developmental stages have been shown to result in insufficient physical activity in adulthood (Telama et al., 2005) and to constitute a central risk factor for both severe somatic and psychiatric diseases (Pedersen and Saltin, 2015). Consequently, the promotion of physical activity in adolescents is a major health issue, e.g., for the prevention of metabolic syndrome and depression (Janssen and Leblanc, 2010; Ahn and Fedewa, 2011). In addition to structured exercise activities (e.g., jogging or playing soccer), an active lifestyle (e.g., walking to school instead of taking the school bus) has been shown to be related to health benefits (Healy et al., 2008; Wen et al., 2011).

To promote a healthy lifestyle, e.g., enhancing non-exercise activity levels in everyday life, an understanding of the psychological determinants of non-exercise activity is necessary. Recent studies have shown associations between mood and physical activity in everyday life, i.e., it has been supposed that mood influences physical activity (Schneider et al., 2009; Liao et al., 2015, 2017a,b), and physical activity has been supposed to influence mood (Kanning and Schlicht, 2010; Schwerdtfeger et al., 2010; Ebner-Priemer et al., 2013; Kanning et al., 2015; Reichert et al., 2017) thus the causality of these effects remains still unknown (Schwerdtfeger et al., 2010; Kanning et al., 2013).

There are several theories supposing possible mechanisms that might explain the associations between mood and physical activity (Thayer et al., 1994; Salovey et al., 2000; Seligman et al., 2005; Stathopoulou et al., 2006; Carels et al., 2007; Mata et al., 2012; Taquet et al., 2016). For example, it is conceivable that people engage in physical activities to increase their low levels of mood (Stathopoulou et al., 2006; Mata et al., 2012; Taquet et al., 2016). This regulation theory (Thayer et al., 1994) suggests that low mood states precede physical activity bouts and make people move. Concurrently, one can hypothesize that people engage in physical activities to facilitate their high mood levels (Carels et al., 2007). This maintenance theory (Salovey et al., 2000; Seligman et al., 2005) suggests that high mood states precede physical activity bouts and make people move.

Technologically speaking, ambulatory assessment makes it feasible to study psychological processes in everyday life. Particularly, assessing mood via e-diaries on smartphones both in real time and in real life and capturing physical activity objectively with accelerometers enables the investigation of how mood and physical activity fluctuate within persons over time (Ortega et al., 2013; Kanning et al., 2015). This real time and real life assessment technique, known as ambulatory assessment or ecological momentary assessment, comes with several advantages such as bypassing laboratory distortions and minimizing well-known recall biases of traditional approaches such as paper-pencil questionnaires (Stone, 2002; Fahrenberg et al., 2007; Bussmann et al., 2009). Additionally, objective physical activity measurements have shown increased validity compared to subjective self-ratings (Prince et al., 2008; Adamo et al., 2009). Most importantly, ambulatory assessment is a state-of-the-art technique that is used to investigate within-subject processes (Trull and Ebner-Priemer, 2013; Kanning et al., 2015). This is of special importance since conclusions from between-subject studies cannot be translated into within-subject processes. For example, at the between-subject level, people who are more physically active on average are the ones with habitually lower mean values of blood pressure, whereas physical activity increases acute blood-pressure at the within-subject level (e.g., bouts of physical activity in everyday life, such as running to the train station increasing a person's blood pressure; Kanning et al., 2013; Reichert et al., 2016a).

In adolescents, there are currently few studies investigating the association of mood and physical activity. Specifically, Schneider et al. (2009) studied the association between affective responses to exercising and physical activity behaviors in the everyday lives of 124 adolescents aged 14–16 years. The participants were asked to perform two ramp-type cycle ergometer exercise tasks. Valence was assessed with an 11-point bipolar feeling scale before, during and after the exercise tasks. Thereafter, the participants wore an Actigraph® accelerometer on their left hips measuring the physical activity levels in their everyday lives. The affective response to the moderate-intensity exercise task was positively associated with physical activity levels in everyday life. However, the affective response to the high-intensity exercise task was not associated with the physical activity in everyday life. These findings led to the assumption that adolescents who show positive affective responses to moderate exercise are more likely to meet activity recommendations in their everyday lives than adolescents who feel uncomfortable with moderate exercise are. Dunton et al. (2014) analyzed the association between mood states and moderate to vigorous physical activity in the everyday lives of 119 children aged 9–13 years. The participants were asked to fill in e-diaries on smartphones for eight consecutive days and to wear accelerometers in their everyday lives. The analyses revealed increased levels of feeling energetic and decreased levels of fatigue before and after 30-min bouts of moderate-to-vigorous physical activity (MVPA) compared to the children's affective baseline levels. Additionally, Dunton et al. (2014) revealed decreased levels of negative affect after 30-min bouts of MVPA. Moreover, children who exercised more often showed a high stability of positive and negative affect during the entire study period. However, the analyses did not show any relation between positive affect and physical activity. Applying a laboratory approach, Subramaniapillai et al. (2016) investigated feelings of tranquility, revitalization, and physical exhaustion prior to and after a 20 min exercise bout on a cycling ergometer in adolescent bipolar patients (*n* = 32) and healthy controls (*n* = 31), both aged 13–20 years. They found a small reduction in feelings of tranquility after exercise in both groups. Langguth et al. (2016) investigated the within-subject association between MVPA and depressed affect in the everyday lives of 72 adolescents aged 13–22 years. The participants wore accelerometers and filled in either online- or paper-pencil-based diaries reporting depressed affect at the beginning and the end of each of the eight consecutive days. Langguth et al. (2016) reported a significant positive association between the mean MVPA level across 1 day and the reported depressed affect the following morning on weekdays in young women. In practice, a 60-min increase in MVPA significantly predicted a 50 percent decrease in next-morning depressed affect. However, this finding was limited to the data provided by women. Moreover, neither women nor men showed any relation between physical activity and affect on weekend days, and physical activity was not related to depressed affect in the evening either. Hulley et al. (2008) investigated the school tendencies of 5–10-year old children and found significantly increased arousal and affective valences in children traveling further distances. They assessed mood by using the children's feeling scale and the children's felt arousal scale and physical activity by a pedometer over a 2-week period in 99 children. Kühnhausen et al. (2013) investigated participants aged 8–11 years (*N* = 82) by applying the ambulatory assessment, i.e., attaching accelerometers and providing e-diaries for several weeks, but did not find any relation between physical activity and affect in everyday life.

In adults, the majority of ambulatory assessment studies on within-subject associations between mood and physical activity have shown significant positive relations between the mood dimension, energetic arousal, and physical activity (Liao et al., 2015; Reichert et al., 2016b). Furthermore, a significant negative relation between calmness and physical activity was found (for an overview, refer to Liao et al., 2015; Reichert et al., 2016b). Findings on the within-subject associations between affective valence and physical activity remain inconclusive (Liao et al., 2015; Reichert et al., 2016b). Recently, Reichert et al. (2017) showed that there may be differential effects of physical activity on mood demonstrating that non-exercise activities (such as climbing stairs) significantly increased energetic arousal and decreased calmness, whereas exercise (such as jogging) significantly increased affective valence and calmness.

While the ambulatory assessment is a state-of-the-art approach for investigating within-subject associations between mood and non-exercise activity (Kanning, 2013), the few existing studies that have utilized this technique in adolescents have shown considerable limitations (Kühnhausen et al., 2013). First, though there are a couple of studies that have focused only on clinical samples (Gawrilow et al., 2016; Subramaniapillai et al., 2016), there have been few studies of healthy participants. Second, most of the existing studies have applied laboratory intervention designs that were concentrated on between-subject effects and, thus, did not reveal findings on the within-subject processes of the everyday lives of children and adolescents. Third, the majority of these studies used self-report assessments that are known to be less reliable than objective physical activity measurements are (Prince et al., 2008). Fourth, recent ambulatory assessment studies in adolescents revealed a lack of power regarding both sample size (between-subject level) and the amount of e-diary ratings (within-subject level). Fifth, parameterization of non-exercise activity is controversial. It is discussed whether it is more feasible to use categories (e.g., light physical activity, moderate to vigorous physical activity, etc.) or to apply a dimensional measurement, such as movement acceleration intensity (Ebner-Priemer et al., 2013; Dunton et al., 2014). Sixth, investigations of the associations between mood and physical activity across different time frames are lacking (Kühnhausen et al., 2013).

Thus, we investigated the association between mood and non-exercise activity in healthy participants to analyze within-subject processes in the everyday lives of adolescents. Furthermore, we conducted an ambulatory assessment study using e-diaries and accelerometers across seven days to study a large community-based sample of healthy adolescents. Non-exercise activity was measured via accelerometers and electronic diaries (e-diaries) on smartphones were applied to repeatedly assess mood (i.e., valence, energetic arousal, and calmness) in real life and real time. Additionally, explorative analyses were conducted to investigate the time frames of physical activity that are associated with the three mood-dimensions of valence, energetic arousal, and calmness.

Based on the findings of existing studies in adolescents and adults (Hulley et al., 2008; Schneider et al., 2009; Kühnhausen et al., 2013; Dunton et al., 2014; Langguth et al., 2016; Reichert et al., 2016b; Subramaniapillai et al., 2016), we hypothesized a positive relationship between the mood dimension, energetic arousal, and subsequent non-exercise activity (hypothesis I). Furthermore, we expected a positive relationship between the mood dimension, valence, and subsequent non-exercise activity (hypothesis II) and a negative relationship between the mood dimension, calmness, and subsequent non-exercise activity (hypothesis III). Moreover, we conducted explorative analyses on the time scale of the effects.

## Materials and methods

### Participants

Adolescents aged between 12 and 17 years were selected during a 29-month period (from April 2014 to January 2017) as part of the URGENCY study (Impact of Urbanicity on Genetics, Cerebral Functioning and Structure and Condition in Young People) at the psychiatric-epidemiological center (PEZ), Central Institute of Mental Health (CIMH), Mannheim, Germany. Participants received monetary compensation for their participation in the study. The exclusion criteria were acute diseases, current or previous cardiovascular disorders, mental disorders, and chronic endocrine or immunological diseases. Participants reported their exercise activities during the study following the same procedure that is described for adults in Reichert et al. (2017). This enabled a focus on the analyses of non-exercise activity through excluding e-diary prompts prior to or during exercise activities. Furthermore, the entire datasets of 6 out of 134 participants were excluded because of missing accelerometer data (devices got lost, recordings were incomplete, etc.); 12 out of 134 of the datasets were excluded for reasons including a large amount of accelerometer non-wear time; and three participants' datasets were excluded because of low e-diary compliance (<30%). Finally, the analyzed sample comprised *N* = 113 participants (48% female) with an average age of 15.02 years (*SD* = 1.70; see Table 1) and an average BMI of 20.14 kg/m^2^ (*SD* = 2.66; see Table 1).

**Table 1:** Descriptive statistics.

	***N***	**Minimum**	**Maximum**	**Mean**	***SD***
Age	113	11.50	17.88	15.02	1.70
BMI (kg/m^2^)	113	14.10	29.40	20.14	2.66
Movement acceleration (mean/participant/week)	113	13.32	74.78	40.86	11.87
Valence (mean/participant/week)^a^	113	4.10	6.95	5.59	0.54
Calmness (mean/participant/week)^a^	113	3.15	6.63	5.13	0.63
Energetic arousal (mean/participant/week)^a^	113	3.27	6.22	4.55	0.65
Compliance (percent/week)	113	42.86	100.00	81.95	14.24
Compliance (per day)	113	5.14	13.43	6.37	0.97

a *Mood (i.e., valence, energetic arousal, and calmness) was assessed on 7-point Likert scales (0–6); for details see Method section*.

### Ambulatory assessment procedure

In the first step, participants were briefed on the usage of both the smartphone (Motorola Moto G) and the accelerometer (Move-II and Move-III) at the PEZ. Afterwards, participants were monitored for seven consecutive days in their everyday lives. A smartphone app (movisensXS, version 0.6.3658) was programmed to trigger e-diary prompts. The daily smartphone-based assessment period lasted from 16:00 to 20:30 on weekdays (Monday to Friday, to not interfere with school duties) and from 9:00 to 20:00 on weekends (Saturday, Sunday). Physical activity was measured continuously except during the night.

On weekdays, participants were asked to answer 4–7 prompts per day. On the weekend, participants were asked to complete 8–17 prompts per day. An acoustic signal, vibration and text on the smartphone invited participants to answer the mood questionnaire. Participants had the opportunity to delay a prompt for 5, 10, or 15 min in unfavorable situations. Since traditional time-based sampling might miss episodes of low and high levels of non-exercise activity (Ebner-Priemer et al., 2013), we used GPS-triggered e-diaries to capture episodes of low and high physical activity in everyday life. Specifically, the GPS trigger released a prompt when passing over a distance of 0.5 km. Additionally, two prompts per day occurred at fixed times (at 16:30 and 20:20). The minimum period between two consecutive prompts was 37 min and the maximum period was 77 min.

Because our analyses were focused on non-exercise activities, we excluded exercise times from our within-subject data. To this end, we asked participants to report on their exercise activities across the study week (i.e., exercise duration and time points) when returning the devices. We applied a well-tried procedure published in (Reichert et al., 2017), which was similar to the Day Reconstruction Method (DRM; 15), used GPS-trajectories tracked via smartphone and involved displays on a digital map to enhance recall.

### Measures

Non-exercise activity was assessed by a triaxial acceleration sensor (move-II or move-III), with a measurement range of ±8 g, a sampling frequency of 64 Hz, a resolution of 12 bits, and that was stored on an internal memory card. Participants wore the devices on their right hips across 7 days during wake hours. The assessment of mood was realized by using an instrument developed by Leonhardt et al. (2016). This instrument, based on the multidimensional mood questionnaire (Wilhelm and Schoebi, 2007), is, to the best of our knowledge, the only existing validated instrument for assessing mood in children and adolescents through e-diaries in real life. Leonhardt et al. (2016) showed this instrument to be appropriate for assessing within-subject dynamics of mood via e-diaries in everyday life. Accordingly, we assessed mood as a three-dimensional construct including good–bad mood, later referred to as valence (items: “cheerful, content, delighted, fantastic, good, afraid, mad, miserable, unhappy”), alertness–tiredness, later referred to as energetic arousal (items: “active, concentrated, interested, exhausted, faint, tired”), and calmness–tension, later referred to as calmness (items: “pleasant, rested, anxious, on edge, stressed”). We presented the items in a mixed order and with reversed polarity using seven point Likert scales.

### Analyses

First, the accelerometer data were processed, i.e., we computed the movement acceleration intensity [mg/min], with the DataAnalyzer (version 1.6.12129) software. Using a high-pass filter (0.25 Hz), gravitational components were excluded, and artifacts were eliminated by using a low-pass filter (11 Hz).

Second, the e-diary data and movement acceleration intensity (mg) data were combined using DataMerger (version 1.6.3868). We defined non-exercise activity as the aggregated mean movement acceleration intensity (mg) over 10-min timeframes, as has been done in previous studies (e.g., Bossmann et al., 2013; Kanning, 2013; Kanning et al., 2013, 2015; Reichert et al., 2016b).

Third, statistical analyses were performed using SPSS (IBM; version 24). The mean movement acceleration intensity (mg) was aggregated for the 10-min intervals after each answered e-diary assessment (later termed as [0–10]). Additionally, the time effects of mood on non-exercise activities were analyzed by aggregating the mean movement acceleration intensity (mg) in the 10-min intervals following each answered e-diary assessment. Specifically, we aggregated the movement acceleration intensity [mg] in timeframes ranging from 11–20, 21–30 and up to 281–290 and 291–300 min (later termed as [11–20], [21–30], etc.). Moreover, we excluded e-diary-assessments that occurred within the 300 min prior to exercise activities (i.e., jogging, playing football, etc.). We log-transformed the outcome variables since the distribution of the non-exercise activity was right-skewed (caused by a high level of sedentary behavior) and showed only few high values (e.g., catching the train). Specifically, we log-transformed all values for the intervals ([0–10], [11–20], [21–30],…up to [291–300]) of the non-exercise activity using the natural logarithm.

Forth, we performed multilevel analyses to identify the within-subject effects of the mood dimensions (i.e., valence, energetic arousal, and calmness) on non-exercise activity using the statistical software, SPSS (version 24, IBM). We nested repeated measurements (i.e., level 1) within participants (i.e., level 2) and calculated intra-class correlation coefficients. The logarithmized values of the non-exercise activity, i.e., the mean movement acceleration intensities in the 10-min intervals after the mood assessments served as depended variable. The level 1 predictors time, time-squared, valence within-subject, energetic arousal within-subject, and calmness within-subject were included. The valence within-subject, energetic arousal within-subject, and calmness within-subject were person mean-centered around the mood scores within the study week. The predictors time and time-squared were transformed, i.e., we subtracted a value of 9 since adolescents received their e-diary prompts at the earliest time of 9:00. We controlled for any between-subject effects by adding the level 2 predictors of age, gender, BMI (kg/m^2^), and exercise/week (min). Additionally, the mean mood scores were added as a level 2 predictor for each participant in terms of the between-subject values for valence, energetic arousal, and calmness. Specifically, to calculate these predictors, we aggregated the mean mood scores for each participant over the whole study week. Random effects were added for every level 1 predictor. However, in the final model (Table 2), only the significant random effects were kept.

**Table 2:** Multilevel model analysis of the influences of the mood dimensions on non-exercise activity: fixed and random effects.

**Outcome**	**Fixed**				**Random**			
**Predictor**	**Beta coefficient**	**Standard error**	***t*-value (*df*)**	***p*-value**	**Variance estimate**	***SD***	**Wald-Z**	***p*-value**
Intercept	3.817	0.745	5.23	<0.001	1.353	0.034	39.673	<0.001
Time (h)	0.351	0.027	13.149	<0.001				
Time-squared (h^2^)	−0.026	0.002	−12.944	<0.001				
Age (years)	−0.0583	0.031	−1.911	0.061				
Gender	−0.095	0.104	−0.914	0.365				
BMI (kg/m2)	−0.033	0.020	−1.635	0.107				
Exercise/week (min)	0.001	0.000	2.105	0.039				
Valence within-subject^a^	0.176	0.035	5.065	<0.001				
Energetic arousal within-subject^a^	0.179	0.037	4.884	<0.001	0.056	0.017	3.226	0.001
Calmness within-subject^a^	−0.164	0.030	−5.409	<0.001				
Valence between-subject^a^	0.263	0.133	1.975	0.053				
Energetic arousal between-subject^a^	−0.014	0.098	−0.145	0.885				
Calmness between-subject^a^	−0.229	0.115	−1.997	0.050				

a *Mood (i.e., valence, energetic arousal, and calmness) was assessed on 7-point Likert scales (0–6); for details see Method section*.

Finally, we computed 30 multilevel models by varying the outcome variable (see **Figure 2**) to focus on either the short- or long-term impacts concerning mood and non-exercise activity. In detail, we incorporated the mean movement acceleration intensities (logarithmized values) aggregated across time frames [0–10], [11–20], [21–30],…up to [291–300]. However, only the level 2 predictors of age, valence between-subject, energetic arousal between-subject, and calmness between-subject were added. Random slopes were not considered for the analyses of time courses, only random intercepts. The α-level for all the analyses was set to 0.05.

#### Model 1: main model

Level 1 equation:

$$\begin{array}{l} Y_{ij}=\;\beta_{0j}+\;\beta_{1j}\times {Time\;of\;the\;day}_{ij}+\;\beta_{2j}\times {Time\;of\;the\;day}_{ij}^{2}\\ +\;\beta_{3j}\times Valence_{ij}+\;\beta_{4j}\times {Energetic\;Arousal}_{ij}+\;\beta_{5j}\\ \times \;Calmness_{ij}+\;r_{ij} \end{array}$$

Level 2 equation:

$$\begin{array}{l} \beta_{0j}=\;\gamma_{00}+\;\gamma_{01}\times Age_{j}+\;\gamma_{02}\times Gender_{j}+\;\gamma_{03}\times BMI_{j}\\ +\;\gamma_{04}{\;\times \;Exercise\;per\;week}_{j}+\;\gamma_{05}\times Valence_{j}\;+\;\gamma_{06}\\ \times Energetic\;Arousal_{j}+\;\gamma_{07}\times Calmness_{j}+u_{0j}\\ \beta_{1j}=\;\gamma_{10}\;+u_{1j}\\ \beta_{2j}=\;\gamma_{20}\\ \beta_{3j}=\;\gamma_{30}\\ \beta_{4j}=\;\gamma_{40}\;+u_{4j}\\ \beta_{5j}=\;\gamma_{50} \end{array}$$

Level 1 reflects the within-subject effects. The subscript *j* refers to participant *j* and the subscript *i* to the time of the e-diary entry. Thus, *Y*_*ij*_ represents the estimated level of non-exercise activity at a given time, *i*, in participant, *j*. The beta coefficients (β) at level 1 represent both the intercept and the effects of the time of the day, the time of the day squared, within-subject valence, energetic arousal, and calmness. *r*_*ij*_ represents the residuals. Level 2 reflects the between-subject effects. As mentioned above, we kept only the random effects showing significance in our final model. Only two predictors, i.e., time of the day and energetic arousal, showed significant random slopes. Therefore, *u*_1*j*_ and *u*_4*j*_ represent the variation in the participants' individual slope estimates for the predictors time of the day and energetic arousal within-subject around the respective overall mean slope estimates (refer to Table 2).

#### Model 2: analyses of time course

We explored the short- and long-term effects of mood on non-exercise activity by calculating 30 multilevel models using different outcome variables, i.e., non-exercise activity within the time frames [0–10], [11–20], [21–30],…up to [291–300] min following each e-diary prompt. We applied the model specified above; however, we simplified the random part by only allowing for variation in the participants' individual intercepts (refer to model equation below).

Level 1 equation:

$$\begin{array}{l} Y_{ij}=\;\beta_{0j}+\;\beta_{1j}\times {Time\;of\;the\;day}_{ij}+\;\beta_{2j}\times {Time\;of\;the\;day\;}_{ij}^{2}\\ +\;\beta_{3j}\times Valence_{ij}+\;\beta_{4j}\times Energetic\;Arousal_{ij}+\;\beta_{5j}\\ \times \;Calmness_{ij}+\;r_{ij} \end{array}$$

Level 2 equation:

$$\begin{array}{l} \beta_{0j}=\gamma_{00}+\gamma_{01}\times Age_{j}+\gamma_{02}\times Exercise\;per\;week_{j}+\gamma_{03}\times Valence_{j}\\ \;+\;\gamma_{04}\times Energetic\;Arousal_{j}+\;\gamma_{05}\times Calmness_{j}+u_{0j}\\ \beta_{1j}=\;\gamma_{10}\\ \beta_{2j}=\;\gamma_{20}\\ \beta_{3j}=\;\gamma_{30}\\ \beta_{4j}=\;\gamma_{40}\\ \beta_{5j}=\;\gamma_{50} \end{array}$$

To make practical conclusions regarding the impacts of changes in mood dimensions on non-exercise activity, we computed percentage change rates using the equation below. A mood-increase of 1 point (mood was rated on Likert-scales from 1 to 7) will lead to a percentage change of non-exercise activity by

$$\delta =\left(\left(e^{\beta \left(valence,\;energetic\;arousal,\;or\;calmness\right)\times 1}\right)\;-\;1\right)\times 100$$

### Ethical considerations

The ethics committee of the Medical Faculty Mannheim at the Heidelberg University approved the study. The ethical guidelines for medical research (i.e., the Declaration of Helsinki) were followed. Information concerning the procedures in the study was communicated to all adolescent participants and their parents before written consent was obtained by their parents. The participants were free to withdraw from the study at any time.

## Results

### Descriptive statistics

On average, the participants answered 45.12 (*SD* = 7.8) mood assessments/participant/week, corresponding to an overall e-diary compliance of 82% (*SD* = 14.23). The participants' mean valence was 5.59 (*SD* = 0.54), their mean energetic arousal was 4.55 (*SD* = 0.65), and their mean calmness was 5.12 (*SD* = 0.63, refer to Table 1). As described above, we defined non-exercise activity as the mean physical activity across the 10-min intervals directly following the e-diary prompts. On average, the participants' non-exercise activity across the whole study period was 40.86 mg/participant/min (range = 13.32–74.78; *SD* = 11.87). For reasons of comparison, it should be noted that according to Anastasopoulou et al. (2014), sedentary behavior (such as sitting) results in approximately 7 mg, walking (3.1 mph gait speed) in approximately 367 mg, and jogging (6.5 mph gait speed) in approximately 1,103 mg. Within-subject variations in non-exercise activity accounted for 69% of the total variance (intra-class correlation coefficient, ρ_*I*_ = 0.041). Sixty-eight participants (76%) engaged in exercise within the study week, on average, for 186.2 min/participant/week (*SD* = 137.8). Since we focused our analyses on non-exercise activity (such as climbing stairs or gardening), we excluded all e-diary prompts if exercise followed in the 300 min following the prompts (which was the case in 9% of all prompts), resulting in a final dataset of 3590 mood assessments.

### Influence of mood on non-exercise activity

Our results revealed significant within-subject associations for each of the three mood dimensions (i.e., valence, energetic arousal, and calmness) with non-exercise activity. Table 2 shows the influences of various within-subject (level 1) and between-subject (level 2) predictors on non-exercise activity, which we parameterized as the 10-min intervals of physical activity directly following each e-diary entry. In detail, energetic arousal showed the expected (hypothesis I) positive, within-subject association with non-exercise activity (beta coefficient = 0.179; *p* < 0.001; refer to Table 2). In practice, a 1 point increase (on a Likert scale from 1 to 7) in energetic arousal led to an average increase in non-exercise activity of around 20% across the 10 min following the e-diary assessment. The mood dimension, valence, was positively correlated with non-exercise activity, as well (beta coefficient = 0.177; *p* < 0.001; refer to Table 2), thus, confirming hypothesis II. In particular, when a participant's valence increased by 1 point (on a likert scale from 1 to 7), their non-exercise activity was increased, on average, by around 19% across the 10-min interval following the e-diary prompt. Calmness showed the expected (hypothesis III) significant association with non-exercise activity (*p* < 0.001; refer to Table 2); however, the effect was in the negative direction (beta coefficient = −0.164; refer to Table 2). In other words, when participants felt 1 point calmer (on a Likert scale from 1 to 7), their subsequent non-exercise activity in the 10 min after the e-diary prompt was decreased by approximately around 15%. In summary, all three mood dimensions showed comparable magnitudes of effects on non-exercise activity (beta coefficient of valence = 0.177, beta coefficient of energetic arousal = 0.179, beta coefficient of calmness = −0.164, refer to Table 2). Moreover, a significant random effect of the mood dimension, energetic arousal, revealed variability in the within-subject association of this mood dimension with non-exercise activity between subjects (beta coefficient = 0.056; *p* = 0.001; refer to Table 2).

**Figure 1:**
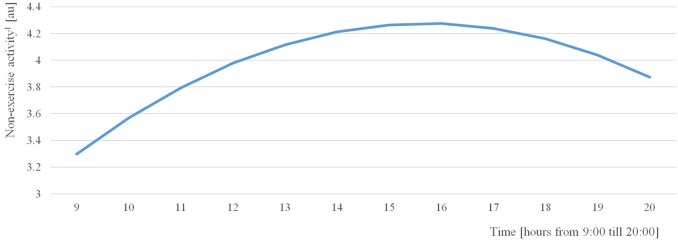
NEA in the course of the day. The daily time effect on non-exercise activity was reversely u-shaped. From the daily study start time (at approximately 9:00) non-exercise activity increased to the afternoon (at approximately 16:00) and decreased then until the study end time (at approximately 20:00). ^1^Arbitrary unit: values are based on milli-g but log-transformed for statistical reasons (for details refer to the methods section).

Additionally, the within-subject predictors, time of day and time of day squared, revealed significant effects on non-exercise activity (beta coefficient of time = 0.351, *p* < 0.001; beta coefficient of time-squared = −0.026, *p* < 0.001). In practice, the effect of the time of day on non-exercise activity was reversely u-shaped, i.e., non-exercise activity increased from the daily study start time (at approximately 9:00) to the afternoon (approximately 16:00) and then decreased until the study end time (at approximately 20:00; see Figure 1). Moreover, the predictor calmness between-subject was significantly associated with non-exercise activity, and the predictor valence between-subject approached significance (*p* = 0.050 and *p* = 0.053, respectively; refer to Table 1). In practice, participants with a higher average valence over the study week showed a higher average amount of non-exercise activity within the 10-min intervals following the e-diary prompts (beta coefficient = 0.263; *p* = 0.053). Similarly, participants feeling, on average, calmer throughout the study week showed lower non-exercise activity levels (beta coefficient = −0.229; *p* = 0.050; refer to Table 2) on average. Furthermore, the amount of exercise (in min) in which adolescents engaged in throughout the study week was significantly related to the non-exercise activity following the e-diary entries. In practice, adolescents who engaged in more exercise within the study week were the ones who were more physically active in their everyday lives, as well. Neither age, body mass index (BMI) nor gender showed an association with non-exercise activity.

### Time course of within-subject associations between mood and non-exercise activity

Within-subject relations between mood and physical activity have mostly been investigated using relatively short periods of physical activity (Kanning and Schlicht, 2010; Kanning, 2013; Kanning et al., 2013, 2015; Dunton et al., 2014; Liao et al., 2017a,b). Only a few studies have investigated the time course of the effects between physical activity and mood (e.g., Schwerdtfeger et al., 2010; Reichert et al., 2016b, 2017). To the best of our knowledge, there has been no investigation of this issue in adolescents. To investigate the time course of within-subject associations between mood and non-exercise activity, we conducted multiple multilevel-analyses in which similar models to our full multilevel model presented above (refer to Table 2) were applied. We incorporated consecutive 10-min timeframes of non-exercise activity following the e-diary entries, i.e., non-exercise activity within 11–20, 21–30,…up to 281–290 and 291–300 min following the e-diary entries, later called [11–20], [21–30], etc.

**Figure 2:**
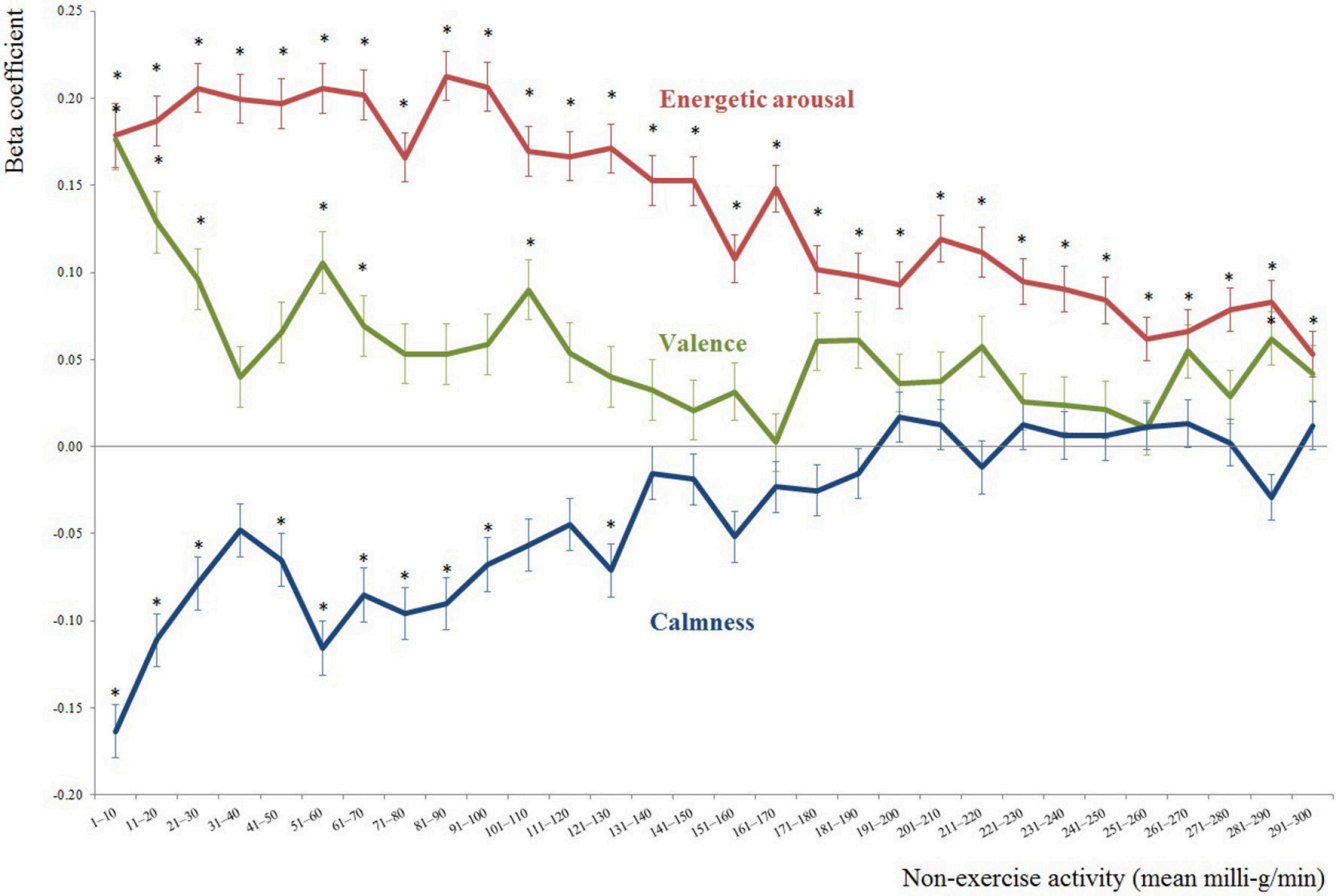
Effects of mood on non-exercise activity aggregated across subsequent 10-min intervals after the e-diary prompt. The beta coefficients for valence, energetic arousal, and calmness predicting non-exercise activity are presented at the y-axis. The x-axis shows the 10-min intervals of non-exercise activity, e.g., the mean non-exercise activity from min 31 up to min 40 after an e-diary prompt is represented by the axis label [31–40]. Significant effects of valence, energetic arousal and calmness predicting 10-min intervals of non-exercise activity are indicated with ^*^ (*p* ≤ 0.05).

On the y-axis of Figure 2, the beta coefficients of the multilevel models are depicted, i.e., each mood dimension (valence, energetic arousal, and calmness) predicting non-exercise activity aggregated across the consecutive 10-min intervals after the e-diary prompt (i.e., 1–10 min; 11–20 min, […], 291–300 min; refer to the x-axis).

For all three mood dimensions, Figure 2 shows the diluting effects over time. In particular, the effects of calmness on non-exercise activity approached zero within the 300-min period after the e-diary prompts; the effects of valence and energetic arousal decreased toward zero, showing the robustness of our significant findings across time. In detail, valence showed significant associations with non-exercise activity only in the first hour after the e-diary prompt and in the timeframes [101–110] and [281–290] (refer to Figure 2). In contrast, the beta coefficients of energetic arousal predicting non-exercise activity showed significance across all timeframes suggesting a strong and stable effect. However, again, the association of energetic arousal with non-exercise activity decreased over time [from [1–10] (beta coefficient = 0.179; *p* < 0.001) to [291–300] (beta coefficient = 0.053; *p* = 0.041)]. This aligned with the findings from our main model, revealing a high influence of energetic arousal on non-exercise activity. Moreover, the mood dimension, calmness, showed a significant relation with non-exercise activity in time frames up to 130 min after the e-diary prompt, again supporting the findings from the main model showing that calmness exerted a negative influence on non-exercise activity. However, looking at the influences of calmness on non-exercise activity over time, some time frames up to 130 min after the e-diary prompt, i.e., [31–40] and [101–120] and the time points onwards, i.e., [131–300], did not reach significance.

## Discussion

We investigated the effects of several mood dimensions (i.e., valence, energetic arousal, and calmness) on non-exercise activity in a community-based sample of healthy adolescents. As expected, our analyses showed significant within-person influences of all three mood dimensions on non-exercise activity.

First, we found a significant, positive within-subject correlation between the mood dimension, energetic arousal, and subsequent non-exercise activity, thus, confirming our hypothesis I. We found indicators of a robust effect of energetic arousal on non-exercise activity, i.e., both a high beta coefficient of energetic arousal predicting non-exercise activity in the 10-min interval following the mood assessment and significant associations between energetic arousal and non-exercise activity across all timeframes up to 300 min after the mood assessments. Indeed, our finding of a significant within-subject correlation between the mood dimension, energetic arousal, and subsequent non-exercise activity was in line with the results from Dunton et al. (2014), who showed that children aged 11–13 years engaged in more moderate to vigorous physical activity following higher ratings of feeling energetic and lower ratings of feeling tired. Although methodologically very different, Hulley et al.'s resultsHulley et al. (2008), revealing that children aged 5–11 years with greater distances between their home and school showed significantly increased felt-arousal-scores, supported our findings as well. Furthermore, our findings were in line with ambulatory assessment studies of adults that showed energetic arousal to be strongly related with non-exercise activity (Schwerdtfeger et al., 2010; Reichert et al., 2016b). As already mentioned above, studies in adolescents investigating the relation between mood and non-exercise activity in daily life do not exist.

Second, we found a positive association between valence and subsequent non-exercise activity at the within-subject level in adolescents, thus confirming our hypothesis II. However, valence appeared to be the mood dimension with the weakest association with non-exercise activity in adolescents. This was evidenced by comparing the beta coefficient to those of the other mood dimensions (i.e., energetic arousal and calmness) and by the time course of the effects across the different timeframes of non-exercise activity after the e-diary prompt. This finding was generally in line with previous studies in adults (for a discussion refer to Reichert et al., 2016b). Interestingly, in adolescents, we found valence to be significantly associated with non-exercise activity in timeframes up to 100 min. In contrast, in adults, Reichert et al. (2016b) found that only the 10-min timeframe after the mood assessment was significantly related with non-exercise activity. This might suppose that the association of valence and non-exercise activity is more pronounced in adolescence compared to adulthood. Further research is needed to substantiate this claim. Moreover, our model showed significant effects of valence on non-exercise activity at the between-subject level. In practice, persons with higher mean valence scores across the study week showed higher non-exercise activity levels, on average, across all 10-min timeframes after their mood assessments within the study week. This is not surprising since a couple of cross-sectional studies revealed that adolescents with higher physical activity levels showed higher levels of well-being (Donaldson and Ronan, 2006).

Third, calmness showed significant, negative within-subject associations with non-exercise activity. In particular, when adolescents felt calmer, they showed decreased levels of non-exercise activity both in the 10 min after the e-diary assessment and in timeframes up to 130 min. Since most of the existing studies have treated mood as a two-dimensional construct, not taking into account the dimension of calmness, there are only a few results on associations of this mood dimension with physical activity (Liao et al., 2015). Reichert et al. (2016b) showed increased calmness to be associated with decreased levels of non-exercise activity within adults, as well. In our model, the negative association between calmness and subsequent non-exercise activity held true for the between-subject level also, i.e., adolescents with higher mean calmness scores across the study week showed lower non-exercise activity levels across all 10-min timeframes after the mood assessment.

The results of our study provide evidence for both the maintenance theory (Salovey et al., 2000; Seligman et al., 2005) and the regulation theory (Thayer et al., 1994). In particular, we found that both increased energetic arousal and increased valence were associated with subsequently increased non-exercise activity supposing that positive mood states made people move thus supporting the maintenance theory (Salovey et al., 2000; Seligman et al., 2005). Apart from that, our data showed that decreased calmness was associated with subsequently increased non-exercise activity supposing that this negative mood state made people move. Therefore, our data does support the regulation theory (Thayer et al., 1994), too.

## Limitations

First, a clear differentiation between non-exercise activity and exercise is important when investigating associations between physical activity and mood since both kinds of physical activity have shown distinct effects on mood (Reichert et al., 2017). We focused the analyses of our study on non-exercise activities (e.g., climbing stairs). Thus, excluding exercise activities (e.g., playing soccer) enabled us to make unambiguous statements on the associations between mood and non-exercise activity in adolescents. However, exercise in general and different exercise intensities in particular, that lead to both distinct behavioral responses (e.g., subjective perception of effort) and physiological responses (e.g., heart rate, lactate concentration), may be associated with distinct mood levels in adolescents. For example, studies in adults showed that physical activities with high intensity lead to decreases in mood (Ekkekakis et al., 2008; Schlicht et al., 2013). Thus, future studies should investigate within-subject associations between mood states and exercise at different intensities in adolescents. Second, our study included adolescents in different stages of biological development (puberty stages), i.e., participants aged 12–17 years. We cannot exclude the possibility that the effects of mood on non-exercise activity might differ among those puberty stages. However, to control for any age effects, we incorporated age as a dimensional predictor in our main model, and found that it showed non-significance. Additionally, we computed the interactions of the mood dimensions with age (as a dimensional variable) predicting non-exercise activity in an explorative manner but found no significant interaction effects. Third, since we were interested in the impacts of mood on non-exercise activities, we parametrized non-exercise activity in 10-min intervals following each e-diary prompt. However, the chronology of our independent variables (i.e., mood-dimensions) and dependent variables (i.e., non-exercise activity) constituted only one aspect of causality (Susser, 1991). This, by no means, proved the causality of our results, e.g., since there may be hidden third variables showing similar timely characteristics to the investigated mood dimensions. Since there have been many studies showing the other direction of effects, i.e., physical activity in everyday life increasing mood (Schwerdtfeger et al., 2010; Dunton et al., 2014; Kanning et al., 2015; Reichert et al., 2017), one might suggest that the relation between physical activity and mood may be circular (Schwerdtfeger et al., 2010). To substantiate this hypothesis, further studies are needed. One approach may be to use ecological momentary interventions, e.g., to specifically alter physical activity levels in everyday life and investigate changes in mood (Myin-Germeys et al., 2016). Fourth, our participants attended school on weekdays. Accordingly, their physical activity patterns were limited during school times (usually pupils are forced to remain seated during classes). Since this artificially limits the variance in non-exercise activity (and pupils were not allowed to use smartphones in school), we prompted the e-diaries on weekdays from 16:00 to 20:30. We explored differences between the associations of the mood dimensions with non-exercise activity, comparing weekdays (16:00 to 20:30) to weekend days (9:00 to 20:00) by applying multilevel interaction analyses. One of the three mood dimensions, i.e., energetic arousal, showed a significantly higher correlation with non-exercise activity on weekend days compared to weekdays. Accordingly, further research is needed, e.g., ambulatory assessments across whole weekdays within adolescents' holidays.

## Conclusion

In our study, we investigated how mood and physical activity fluctuate within adolescents in everyday life over time. This understudied association may play an important role in understanding what drives physical activity levels in developmental stages of puberty and is of special importance particularly since physical activity in adolescence predicts physical activity in adulthood (Telama et al., 2005). Applying sophisticated ambulatory assessment procedures, i.e., repeated GPS-triggered mood assessments in real time and objective physical activity measurements in the everyday life of a community-based sample comprising 113 participants aged 12–17 years, we found both valence and energetic arousal to be positively related to subsequent non-exercise activity and calmness to be negatively related to subsequent non-exercise activity. In practice, when adolescents felt better and content, more awake and full of energy, or less calm and relaxed, they engaged more in non-exercise activity within the subsequent timeframe. These findings may help to understand the psychological correlates of physical activity in adolescents' everyday lives, thereby, possibly contributing to the facilitation of physical activity and its beneficial health-promoting effects on the developmental stages of puberty. However, further research is needed, e.g., to unravel the relevant neurobiological foundations and to develop useful practical interventions.

## Author contributions

EK, HT, UB, GG, MG, IR, AZ, AM-L, UE-P, and MR made substantial contributions to the conception and the design of the study. MR and MG acquired data. EK, HT, IR, AM-L, UE-P, and MR analyzed and interpreted the data. EK, HT, UB, GG, MG, IR, AZ, AM-L, UE-P, and MR were involved in drafting the manuscript and revising it critically for important intellectual content. EK, HT, UB, GG, MG, IR, AZ, AM-L, UE-P, and MR have given final approval of this version of the manuscript to be published. EK, HT, UB, GG, MG, IR, AZ, AM-L, UE-P, and MR agree to be accountable for all aspects of the work in ensuring that questions related to the accuracy or integrity of any part of this work are appropriately investigated and resolved.

### Conflict of interest statement

AM-L has received consultant fees from American Association for the Advancement of Science, Atheneum Partners, Blueprint Partnership, Boehringer Ingelheim, Daimler und Benz Stiftung, Elsevier, F. Hoffmann-La Roche, ICARE Schizophrenia, K. G. Jebsen Foundation, L.E.K. Consulting, Lundbeck International Foundation (LINF), R. Adamczak, Roche Pharma, Science Foundation, Sumitomo Dainippon Pharma, Synapsis Foundation—Alzheimer Research Switzerland, System Analytics, and has received lectures fees including travel fees from Boehringer Ingelheim, Fama Public Relations, Institut d'Investigacions Biomèdiques August Pi i Sunyer (IDIBAPS), Janssen-Cilag, Klinikum Christophsbad, Gandampöppingen, Lilly Deutschland, Luzerner Psychiatrie, LVR Klinikum Dandampüsseldorf, LWL PsychiatrieVerbund Westfalen-Lippe, Otsuka Pharmaceuticals, Reunions i Ciencia S. L., Spanish Society of Psychiatry, Sandampüdwestrundfunk Fernsehen, Stern TV, and Vitos Klinikum Kurhessen. The other authors declare that the research was conducted in the absence of any commercial or financial relationships that could be construed as a potential conflict of interest.
